# The effect of pedunculopontine nucleus deep brain stimulation on postural sway and vestibular perception

**DOI:** 10.1111/ene.12947

**Published:** 2016-01-23

**Authors:** N. Yousif, H. Bhatt, P. G. Bain, D. Nandi, B. M. Seemungal

**Affiliations:** ^1^ Division of Brain SciencesImperial College LondonLondonUK

**Keywords:** balance, deep brain stimulation, sensory integration, sway, vision

## Abstract

**Background and purpose:**

Deep brain stimulation (DBS) of the pedunculopontine nucleus (PPN) reduces the number of falls in patients with Parkinson's disease (PD). It was hypothesized that enhanced sensory processing contributes to this PPN‐mediated gait improvement.

**Methods:**

Four PD patients (and eight matched controls) with implanted bilateral PPN and subthalamic nucleus DBS electrodes were assessed on postural (with/without vision) and vestibular perceptual threshold tasks.

**Results:**

Pedunculopontine nucleus ON stimulation (compared to OFF) lowered vestibular perceptual thresholds but there was a disproportionate increase in the normal sway increase on going from light to dark.

**Conclusions:**

The disproportionate increased sway with PPN stimulation in the dark may paradoxically improve balance function since mechanoreceptor signals rapidly adapt to continuous pressure stimulation from postural akinesia. Additionally, the PPN‐mediated vestibular signal enhancement also improves the monitoring of postural sway. Overall, PPN stimulation may improve sensory feedback and hence balance performance.

## Introduction

Parkinson's disease (PD) standard medical (l‐DOPA) and surgical therapy [subthalamic nucleus (STN) deep brain stimulation (DBS)] are effective in reducing patients’ bradykinesia, rigidity and rest tremor but are less successful in controlling postural dysfunction 1. Recent data suggest that pedunculopontine nucleus (PPN) DBS may improve balance function in PD 2. Recent single‐neurone primate data suggest the PPN is highly vestibular‐responsive 3. It was hypothesized that PPN‐related postural improvement may relate to improved sensory processing.

## Methods

Four PD patients (Table 1) with simultaneously implanted bilateral PPN and STN electrodes (males, mean age 61.5 ± 3 years) and eight healthy age‐matched controls (mean age 65 ± 10 years) were recruited. The patients were part of a double‐blind randomized controlled trial comparing the effect of simultaneous STN and PPN DBS to that of STN DBS alone. The average location of the active PPN contacts were 4.5 ± 2.3 mm lateral (perpendicular to midline), −0.1 ± 1.9 mm AP (in relation to PC) and vertical −17.5 ± 1.9 mm (perpendicular to the AC−PC plane 4). Written informed consent was obtained from all participants and the experimental protocol was approved by the local research ethics committee. Participants performed a balance and a vestibular threshold task in counterbalanced order. Patients carried out each task once with PPN stimulation OFF and once with PPN stimulation ON. Patients were blinded to their stimulation setting and the order of PPN stimulation was randomized. Patients remained ON STN stimulation and normal dopaminergic medication throughout.

**Table 1:** Patient demographics including gender, age and unified Parkinson's disease rating scale (UPDRS) scores for the activities of daily living section (II) and the motor examination (III) ON and OFF PPN stimulation

Patient	Gender	Age at testing (years)	Height (cm)	Weight (kg)	Months since DBS	UPDRS				Left PPN settings (at 60 μs)	Right PPN settings (at 60 μs)
						ON		OFF			
						II	III	II	III		
a	M	58	170	76	13	24^a^	48^a^	31	75	1–2+, 1.8 V, 30 Hz	9–10+, 1.8 V, 30 Hz
b	M	64	185	80	6	10	26	24	52	0–1+, 1 V, 20 Hz	8–9+, 1 V, 20 Hz
c	M	65	170	76	21	20	16	30	47	1–2+, 1.6 V, 20 Hz	10+11−, 1.6 V, 20 Hz
d	M	59	173	89	33	13	24	35	45	1–2+, 0.6 V, 20 Hz	9–10+, 0.6 V, 20 Hz

a As one patient was unable to complete assessment with PPN OFF, scores when all stimulation was OFF are shown.

A previously described vestibular threshold task was used 5. Patients sat in a motorized rotating chair in darkness with white noise masking and were required to indicate their direction of motion (left/right). An automated staircase algorithm determined subjects’ perceptual threshold. An average of four trials was obtained.

A force plate (0R6‐5‐1, AMTI, Watertown, MA, USA, 91 × 61 × 17 cm, sampling rate 1000 Hz and calibrated using a 10.2 kg weight on two locations) assessed postural sway by detecting the amount of pressure applied by each foot under two conditions for 120 s: eyes open (EO) and eyes closed (EC) in counterbalanced order. Participants were told to stand with their arms hanging loosely by their sides with their heels 8 cm apart.

Differences between groups were tested using *t* tests (at significance level 0.05); however, due to the small number of patients (*n* = 4), statistical tests were not performed within this group. The Romberg coefficient (RC = sway EO/sway EC) was calculated for participants. An RC = 1 indicates that vision does not affect sway whereas RC < 1 indicates a visual influence on sway since there is greater sway in the dark (EC).

## Results

**Figure 1:**
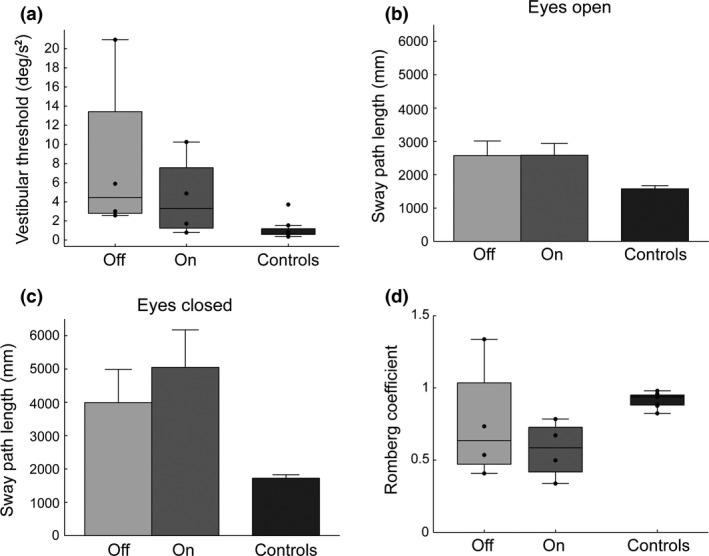
(a) Box plot of vestibular thresholds for the patients and controls. Bar plot of overall sway in the eyes open (b) and eyes closed (c) conditions (EO and EC). (d) The Romberg coefficient (EO/EC) is shown to compare sensory conditions directly.

When PPN stimulation was off, patients had significantly worse (i.e. higher) vestibular thresholds (*t*_10_ = −2.355, *P* = 0.04) compared to controls (Fig. 1a). PPN stimulation lowered vestibular thresholds such that the difference compared to controls was no longer significant (*t*_10_ = −2.136, *P* = 0.06).

Patients displayed significantly more sway compared to controls with eyes open both ON (*t*_10_ = −3.069, *P* = 0.012) and OFF (*t*_10_ = −3.599, *P* = 0.005) stimulation (Fig. 1b) and with eyes closed on (*t*_10_ = −2.584, *P* = 0.027) and OFF (*t*_10_ = −3.016, *P* = 0.0013) stimulation (Fig. 1c). Interestingly, with EO sway was no different whether PPN stimulation was ON or OFF (Fig. 1b), whereas with EC sway increased with stimulation ON compared to OFF (Fig. 1c).

In all groups, RC < 1 indicated more sway with EC (Fig. 1d). OFF stimulation, patients’ RCs were not different from controls (*t*_10_ = 1.171, *P* = 0.269); however, ON stimulation, patients’ RCs were lower than controls (*t*_10_ = 4.870, *P* = 0.001). Thus when PPN stimulation is ON compared to OFF, patients swayed disproportionately more with EC, but there was little change in sway with EO when PPN was ON or OFF.

## Discussion

It was hypothesized that PPN DBS improves postural control in PD patients by enhancing sensory processing.

It was found that, in four patients with PD, PPN DBS improved vestibular perceptual thresholds, supporting recent primate data showing that PPN neurones are vestibular‐responsive. Our patients always had STN stimulation ON and this may support the idea that simultaneous STN and PPN stimulation act synergistically as suggested recently 6. Hence if PPN is a brainstem centre for vestibular processing, its stimulation may improve postural function in PD patients by modulating vestibular signalling.

Despite its purported beneficial effects upon postural control, PPN stimulation paradoxically increased sway in the dark, which could imply worse postural control. However, cutaneous mechanoreceptors in the glabrous skin of the foot, which play a role in postural control 7, are rapidly adapting 8. Hence, excessive rigidity as in the OFF condition will lead to a loss of input from these cutaneous receptors. This mechanoreceptor adaptation can be avoided by increasing sway. It follows that increasing sway above an excessively rigid baseline in PD patients (e.g. with PPN DBS) will maintain mechanoreceptor input for postural control. That improved vestibular thresholds were found, indicating a more reliable vestibular signal, may also enable better monitoring by the postural system of the body's position in space relative to gravity. This improvement in vestibular signalling may thus enable the postural system to safely accommodate any PPN‐related increased sway. Finally, increased sway will provide additional input to the vestibular system and further reduce the uncertainty regarding the estimate of body‐in‐space position.

In conclusion, the improved reliability of the vestibular signal function with PPN stimulation may facilitate a strategy of increased postural movement which further improves sensory feedback by enhancing somatosensory signalling. This prediction of enhancing somatosensory signalling will require specific testing.

## Disclosure of conflicts of interest

The authors declare no financial or other conflicts of interest.
